# Associated factors for depression, suicidal ideation and suicide attempt among asthmatic adolescents with experience of electronic cigarette use

**DOI:** 10.18332/tid/127524

**Published:** 2020-10-16

**Authors:** Chang Woon Kim, Seung Chan Jeong, Joo Young Kim, Ju Suk Lee, Jun Hwa Lee, Seon Hui Jo, Sung Hoon Kim

**Affiliations:** 1 Department of Obstetrics and Gynecology, Samsung Changwon Hospital, Sungkyunkwan University School of Medicine, Changwon, Republic of Korea; 2 Department of Urology, Samsung Changwon Hospital, Sungkyunkwan University School of Medicine, Changwon, Republic of Korea; 3 Department of Pediatrics, Samsung Changwon Hospital, Sungkyunkwan University School of Medicine, Changwon, Republic of Korea; 4 Department of Biostatistics, Samsung Changwon Hospital, Sungkyunkwan University School of Medicine, Changwon, Republic of Korea

**Keywords:** adolescents, asthma, depression, electronic cigarettes, suicide

## Abstract

**INTRODUCTION:**

While electronic cigarette (EC) use is rapidly increasing among asthmatic adolescents, little is known about the links between EC use and depression or suicidality. We assessed associated factors for depression and suicidality in asthmatic adolescents with experience of EC use.

**METHODS:**

We analyzed the data from the 11th to 13th Korea Youth Risk Behavior Web-based Surveys, which were completed from 2015 to 2017. Data were obtained from a stratified, multistage, clustered sample. Students supplied ‘yes or no’ answers to questions about previous asthma diagnosis by a doctor. Associated factors for depression and suicidality were evaluated by logistic regression models after controlling for potential confounding factors. We targeted 203336 adolescents, and 195847 completed the survey.

**RESULTS:**

The proportion of asthma among the respondents was 8.9%. The rate of experience of EC use was higher among asthmatic respondents than non-asthmatic respondents (10.3% vs 8.6%). Asthmatic respondents with experience of EC use had a much higher proportion of negative mental health states including depression and suicidality than subjects without EC experience. In our adjusted models, perception of stress was most strongly associated with depression (adjusted odds ratio, AOR=4.79; 95% CI: 4.12–5.58), and perception of unhappiness was most strongly associated with suicidal ideation (AOR=5.24; 95% CI: 4.51–6.09) and suicide attempt (AOR=4.37; 95% CI: 3.36–5.69).

**CONCLUSIONS:**

Many Korean asthmatic adolescents with experience of EC use report relatively high depression and suicidal behaviors. A multidisciplinary approach, including psychological help, may be required to prevent suicide among this population, especially those who report associated factors.

## INTRODUCTION

Asthma is one of the major chronic respiratory conditions affecting adolescents worldwide, and the increasing prevalence of asthma has become a major public health concern^1^. It is well known that cigarette smoking worsens asthma^2^. While many smokers want to quit and many efforts to help people quit smoking have been implemented, few are able to quit successfully^3,4^. In response, the electronic cigarette (EC) has been marketed as a tobaccobased harm-reduction product that offers hope for adult smokers to give up or reduce their cigarette smoking^5^. ECs provide the sensation of smoking and the desired nicotine effect without burning tobacco^6,7^. The EC market-share has grown rapidly and globally, particularly among smokers and adolescents^3,8^. ECs have become the most common tobacco/nicotine product among US high school students, with a 10-fold increase in the prevalence of past 30-day use (from 1.5% in 2011 to 16.0% in 2015)^9,10^. Several recent studies have indicated that ECs are perceived positively among adolescents, and the rate of EC use is higher among asthmatic adolescents than nonasthmatic adolescents^1,6,11-13^.

In accordance with the dramatic rise in EC use, the central issues of concern are: 1) can EC aid smoking cessation, 2) do young EC users become future combustible tobacco smokers, and 3) what are the health effects among dual users^14,15^. Although there is still very little known about the impact of ECs on asthma in adolescents^3^, of the nearly 50 conclusions in the National Academies of Sciences’ (NAS) report reviewing more than 800 research papers on EC, the substantial or conclusive evidence is the following:

1. EC is less hazardous for smokers seeking to quit than combustible cigarettes, while even more harm-creating for former or never smokers^14-16^. Many studies have commented with necessary emphasis on the uncertainties about safety and adverse effects from the use of EC such as vomiting, nausea, headache, dizziness, eye irritation, lethargy, and harm and death by direct e-liquid contact^5,8,10,17-20^. EC aerosol contains heavy metals such as nickel, tin and lead and other toxic and carcinogenic substances (e.g. formaldehyde, acrolein)^4,5,14^. Although they are generally at levels lower than those of combustible cigarettes, they can induce oxidative stress and acute endothelial cell dysfunction, and then serious lung disease^4,5,7,14,15^.
2. Particularly among youth, EC use appears to increase the risk of future use of combustible tobacco^14,15^. On the point that EC use can serve as a gateway to conventional cigarettes^10^, there is strong evidence for an association between adolescent EC use among never smokers and subsequent smoking initiation^3,8,10,21^.
3. The effectiveness of ECs as smoking cessation devices, the health benefits among dual users, long-term health effects for EC users and the effects of maternal use on fetal development are unknown or unproven^14,15^. However, ECs not only have a negative impact on the users’ cardiovascular health, and are a potential risk for bronchial obstruction and obstructive lung disease, they also have a higher risk for psychological distress such as depression and addictive behaviors in users than in non-smokers^20,22,23^. In addition, particularly with reference to association of smoking (nicotine addiction) and suicidal behavior in adults and adolescents^20,24,25^, it is necessary to actively intervene in adolescents’ smoking to prevent or reduce suicidal behavior.

Despite the fact that suicide is a major critical public health issue and one of the leading causes of death among adolescents in many countries^26^, discussions of depression and suicidality have focused on conventional cigarette use or secondhand smoke exposure^24,25^, and only recently several investigations of the association of depression and suicidality with EC use have been conducted^20,22,23,27,28^. However, the associated factors for depression and suicidality among asthmatic adolescents with experience of EC use, have not been evaluated using a nationally representative sample. Given this background, because more effective prevention of suicide requires a comprehensive understanding of sociodemographic, psychiatric, and somatic risk factors for suicide^26^, we wished to identify the associated factors for depression, suicidal ideation and suicide attempt among asthmatic adolescents with experience of EC use. Thus, we hypothesize that the rate of experience of EC use is greater among asthmatic adolescents than non-asthmatic adolescents based on the results of the aforementioned studies^1,6,11-13^. Among adolescents with asthma, subjects with experience of EC use are more likely to experience mental health problems, including suicidality, than those without EC experience. This study has three aims. First, we aim to describe and compare participants’ characteristics including mental health status of Korean adolescents with and without asthma. Second, among asthmatic adolescents, we wish to investigate and compare characteristics including depression and suicidality according to experience of EC use. Third, among asthmatic adolescents with experience of EC use, we attempt to identify the associated factors for depression, suicidal ideation and suicide attempt, which is the main purpose of our study.

## METHODS

### Study design and groups

This study was designed as a secondary analysis of data obtained from the 11th to 13th wave of the Korea Youth Risk Behavior Web-based Surveys (KYRBSs), completed from 2015 to 2017. The KYRBS, which was first conducted in 2005 by the Korea Centers for Disease Control and Prevention (KCDC), is an annually conducted, cross-sectional, self-reported, question-based online survey of a nationally representative sample of Korean adolescents. The purpose of the KYRBS is to investigate numerous health-risk behaviors among Korean youths aged 12–18 years (from students of the first year of middle school to those of the third year of high school in the Korean education system). It is known that the reliability estimates for the KYRBSs questionnaire show good validity^29^. All KYRBS data analyses were conducted in accordance with KCDC guidelines and regulations. The KYRBS obtains data using a stratified three-stage random cluster sampling method. In the first stage (stratification), the study population was stratified by geographical region (43 area clusters including metropolitan, medium or small cities, or rural areas) and school type (public or private, co-educational, and vocational school) to minimize sampling errors. In the second stage (sample allocation), approximately 75000 students from 400 middle schools and 400 high schools were selected by proportional sampling according to target population composition. In the third stage (stratified cluster sampling), the sample schools were selected using systematic sampling, and sample classes were then selected by simple randomized sampling from selected schools. Stratification occurs on an annual basis, so that different schools are selected each year. Students participate in the KYRBS voluntarily, completing surveys by logging on to survey webpages in school computer rooms with a randomly assigned unique identification number. Anonymity is guaranteed. Following sufficient explanation of the process by teachers, completion of the survey requires usually 45–50 minutes. The online system does not accept non-responses, thus, participants cannot skip to subsequent questions without providing complete consecutive answers to all questions. Parental or legal guardian permission is obtained prior to survey administration, and the consent procedure was approved by the Institutional Review Board (IRB) of the KCDC. Survey participation excluded age-eligible respondents with absenteeism (school absence without permission), special educational needs (such as developmental disabilities), and dyslexia. We obtained data from the 2015–2017 KYRBS datasets from their publicly available website (https://www.cdc.go.kr/yhs/home.jsp). Reasons for non-participation are not available. The protocol for this study was reviewed and approved by the IRB of our hospital (IRB no. 2020-04-009), which also waived the requirement for informed consent because our study is based on secondary analyses of publicly available anonymous data.

### Evaluation indices

#### Asthma

Participants were asked about their history of doctordiagnosed asthma. Lifelong diagnosis of asthma was determined by answering ‘yes’ to the following question: ‘Have you ever been diagnosed with asthma by a doctor at any point in your life?’. Disease severity, current symptoms, symptom management, and treatment modality, were not assessed.

#### Socioeconomic and demographic characteristics

Information on socioeconomic characteristics including age, sex, school grade (middle and high school), perceived socioeconomic status (SES), and academic achievement was gathered. For academic achievement and SES, participants were asked: ‘During the past 12 months, how would you rate your academic performance and your family's SES, respectively?’. Responses were divided into five categories in the survey and we further categorized as high (high or middle-high), middle (middle), or low (middle-low or low) levels depending on the intensity of the response.

#### Smoking and drinking

Smoking status at the time of the study was assessed with the following question: ‘Have you ever smoked cigarettes, even once?’. If the answer was ‘yes’, another question was asked: ‘How many days did you smoke even one cigarette during past 30 days before this survey?’. Those who responded ‘more than one day’ were defined as current smokers by the standpoint of the KYRBS. For alcohol consumption status, participants were asked: ‘Have you ever consumed alcohol, even once?’. If the answer was ‘yes’, the following question was asked: ‘How many days did you drink at least one shot of alcohol during past 30 days before this survey?’. Those who responded ‘more than one day’ were classified as current drinkers. To measure EC smoking, participants were asked a binary (yes or no) question regardless of conventional smoking status or experience: ‘Have you ever used ECs in your lifetime?’. For those who answered ‘yes’, a follow-up question was asked: ‘In the last 30 days, have you used ECs?’. For the main reasons for EC use, participants chose only one answer among several examples such as appealing flavors, safety, or curiosity.

#### Emotional states and suicidality

Subjective healthiness was assessed with the following question: ‘What do you think about your health state?’ with response options of ‘healthy’ (very healthy, healthy), ‘average’, and ‘unhealthy’ (a little unhealthy, very unhealthy). Perceived level of happiness was assessed with a self-rating happiness scale, and responses included ‘happy’ (very happy, a little happy), ‘average’, and ‘unhappy’ (a little unhappy, very unhappy). Perceived stress status was assessed with the following question: ‘How often do you feel stress?’ with response options of ‘often’ (very often, often), ‘sometimes’, and ‘never’ (rarely, never). Levels of sleep satisfaction were assessed by the following question: ‘What is your level of your satisfaction with fatigue recovery after sleep during the last week?’, with score on a 5-point scale, including answers of ‘plenty’ (plenty, enough), ‘a little’, and ‘not enough’ (not enough, never enough). Experience with a depressive mood was defined as a participant reporting feelings of sadness or desperation to any degree that caused cessation of their usual activities almost every day for two weeks or more, within the 12 months preceding the survey. Measures of suicidal ideation and suicide attempt were recorded as binary dependent variables (yes or no), based on responses to the following questions: ‘During the past 12 months, have you ever seriously thought of committing suicide?’ and ‘Have you attempted suicide in the previous 12 months?’. Repetitions of depressive mood, suicidal ideation and suicide attempt were not assessed.

### Statistical analyses

We conducted all statistical analyses using complex sample procedures from the Statistical Package for the Social Sciences (SPSS) software program (ver. 21.0, IBM Corp., Armonk, NY, USA). Because KYRBS data were collected through a representative, stratified, and clustered sampling method, survey data were weighted for statistical representation of the general population based on the sample design. Descriptive statistics were used to describe the basic characteristics of the study population, and numbers and percentages were reported for each variable. Chi-squared tests for categorical variables and independent t-tests for continuous variables were used to compare subjects with and without EC experience among asthmatic adolescents. To identify the associated factors for depressive mood and suicidality among asthmatic adolescents with experience of EC use, we performed multivariate logistic regression analysis after selecting significant covariates. Odds ratios (ORs), adjusted ORs (AORs), and 95% confidence intervals (95% CIs) were obtained via: 1) simple logistic regression with complex sampling (unadjusted), and 2) multiple logistic regression with complex sampling adjusted for age, sex, grade, SES, academic achievement, drinking, smoking, and mental health variables. For all analyses, we considered a p<0.05 to be statistically significant.

## RESULTS

### Participant demographic characteristics according to presence of asthma

Over a 3-year study period, 203336 adolescents were targeted and 195847 completed surveys, yielding a response rate of 96.3%. Table 1 shows the general characteristics and differences between asthmatic and non-asthmatic respondents. The proportion of asthma among the overall respondents was 8.9% (8.8 % in 2015, 9.1% in 2016, and 8.8% in 2017). Asthmatic respondents included higher proportions of males, middle school students, high SES and academic achievement, current drinkers, and smokers, compared with non-asthmatic respondents. Asthmatic subjects perceived themselves as unhealthier and unhappier, having more stress, feeling more unsatisfied with sleep, having depressive moods, and having more frequent suicidal ideation and suicide attempt than subjects without asthma.

**Table 1:** General characteristics according to the presence of asthma among Korean adolescents, 2015–2017 (N=195847)

*Characteristics*	*Asthma*		*p*
	*No (n=178444)*	*Yes (n=17403)*	
**Sex**			<0.001
Female	87912 (48.53)	7304 (41.11)	
Male	90532 (51.47)	10099 (58.89)	
**Grade**			<0.001
Middle school	88491 (45.73)	8912 (47.77)	
High school	89953 (54.27)	8491 (52.23)	
**Subjective economic state**			<0.001
High	66818 (37.78)	6758 (39.16)	
Middle	83965 (46.83)	7635 (43.62)	
Low	27661 (15.38)	3010 (17.22)	
**Academic achievement**			<0.001
High	68428 (38.10)	7025 (40.09)	
Middle	50663 (28.51)	4725 (27.35)	
Low	59353 (33.38)	5653 (32.57)	
**Current drinking**			<0.001
No	151300 (84.21)	14500 (82.80)	
Yes	27144 (15.79)	2903 (17.20)	
**Current smoking**			<0.001
No	166967 (93.30)	15976 (91.69)	
Yes	11477 (6.70)	1427 (8.31)	
**Subjective healthiness**			<0.001
Healthy	130556 (72.90)	11147 (63.98)	
Average	37723 (21.29)	4515 (26.03)	
Unhealthy	10165 (5.81)	1741 (9.98)	
**Subjective happiness**			<0.001
Happy	119642 (66.55)	11200 (63.68)	
Average	45491 (25.84)	4483 (26.26)	
Unhappy	13311 (7.61)	1720 (10.06)	
**Perceived stress**			<0.001
Often	64439 (36.19)	7124 (40.92)	
Sometimes	77005 (43.41)	7136 (41.14)	
Never	37000 (20.40)	3143 (17.94)	
**Sleep satisfaction**			<0.001
Plenty	48569 (26.42)	4599 (25.74)	
A little	58127 (32.59)	5421 (31.32)	
Not enough	71748 (40.99)	7383 (42.94)	
**Depression**			<0.001
No	135469 (75.77)	12337 (70.66)	
Yes	42975 (24.23)	5066 (29.34)	
**Suicidal ideation**			<0.001
No	157816 (88.38)	14740 (84.68)	
Yes	20628 (11.62)	2663 (15.32)	
**Suicide attempt**			<0.001
No	174273 (97.67)	16748 (96.18)	
Yes	4171 (2.33)	655 (3.82)	
**Experience of EC use**			<0.001
No	163851 (91.44)	15635 (89.68)	
Yes	14593 (8.56)	1768 (10.32)	
**Current EC use**			<0.001
No	173270 (97.10)	16744 (96.21)	
Yes	5174 (2.90)	659 (3.79)	

Numbers represent frequency and column percentages for each characteristic. Current smokers were defined as those who responded ‘more than one day’ to the following question: ‘How many days did you smoke even one cigarette during past 30 days before this survey?’ by the standpoint of the Korea Youth Risk Behavior Web-based Surveys. To measure EC smoking participants were asked a binary (yes or no) question regardless of smoking status or experience: ‘Have you ever used ECs in your lifetime?’. For those who answered yes, a follow-up question was asked: ‘In the last 30 days have you used ECs?’. Other definition or concept of variables are mentioned in the Methods section of the manuscript. Survey data were weighted for statistical representation of the general population based on the sample design. Chi-squared test was applied to determine statistical differences between categorical data and independent t-test was used for continuous variables. EC: electronic cigarette.

### Smoking status

Experience of EC use and current EC use were more common among asthmatic adolescents than nonasthmatic adolescents (10.3% vs 8.6%, and 3.8% vs 2.9%, respectively; Table 2). Among asthmatic respondents, current smokers were more prevalent than current EC users (8.3% vs 3.8%; Table 1). Most asthmatic adolescents with experience of EC use (1768 respondents) began using ECs in middle and high school (54.9% and 30.6%, respectively; Table 2). The beginning time for first EC before middle school period happened in 254 (14.5%) participants. The main reasons for EC use were appealing flavors (21.0%), safety (20.5%), curiosity (20.0%), and as a smoking cessation aid (11.3%).

**Table 2:** Beginning time and reasons of using electronic cigarettes among Korean asthmatic adolescents with experience of electronic cigarette use, 2015–2017 (N=1768)

*Beginning time of first EC use*	*Number (weighted %)*
Before elementary school	111 (6.35)
Elementary school	143 (8.18)
Middle school	960 (54.92)
High school	534 (30.55)
***Main reason of EC use***	***Number (weighted %)***
Safety	363 (20.53)
Smoking cessation aid	200 (11.31)
Indoor smoking	177 (10.01)
Easy access	43 (2.43)
Appealing flavors	371 (20.99)
No smell of conventional smoking	127 (7.18)
Curiosity	353 (19.97)
Other	134 (7.58)

For the main reasons for electronic cigarettes, participants chose only one answer among several examples. EC: electronic cigarettes.

### Differences according to electronic cigarette experience among asthmatics

Table 3 shows the general subject characteristics stratified by EC experience among adolescents with and without asthma. The differences between asthma and non-asthma group, including depression and suicidality, according to EC experience, had a similar pattern. Among asthmatic adolescents, subjects with experience of EC use were more likely to be male (81.4%), be enrolled at high school (72.5%), and have a lower academic achievement and SES. Asthmatic adolescents with experience of EC use included higher proportions of current drinkers and smokers, self-perception as unhealthier and unhappier, stress, lack of satisfaction with sleep, depressive moods, and frequent suicidal ideation and suicide attempt than asthmatic adolescents with no EC experience. The prevalence of depression, suicidal ideation and suicide attempt among asthmatic adolescents with experience of EC use were 41.8%, 22.4%, and 10.7%, respectively.

**Table 3:** Differences according to electronic cigarette experience among Korean participants without asthma and with asthma, 2015–2017

*Characteristics*	*Adolescents without asthma (N=178444)*			*Adolescents with asthma (N=17403)*		
	*Experience of EC use*			*Experience of EC use*		
	*No (n=163851)*	*Yes (n=14593)*	*p*	*No (n=15635)*	*Yes (n=1768)*	*p*
**Sex**			<0.001			<0.001
Female	85208 (51.40)	2704 (17.94)		6961 (43.70)	343 (18.62)	
Male	78643 (48.60)	11889 (82.06)		8674 (56.30)	1425 (81.38)	
**Grade**			<0.001			<0.001
Middle school	84734 (47.83)	3757 (23.38)		8391 (50.10)	521 (27.49)	
High school	79117 (52.17)	10836 (76.62)		7244 (49.90)	1247 (72.51)	
**Subjective economic state**			<0.001			<0.001
High	61699 (37.99)	5119 (35.55)		6097 (39.39)	661 (37.18)	
Middle	77744 (47.25)	6221 (42.41)		6993 (44.47)	642 (36.26)	
Low	24408 (14.76)	3253 (22.04)		2545 (16.15)	465 (26.56)	
**Academic achievement**			<0.001			<0.001
High	64509 (39.18)	3919 (26.57)		6513 (41.33)	512 (29.27)	
Middle	47098 (28.89)	3565 (24.47)		4343 (27.99)	382 (21.79)	
Low	52244 (31.93)	7109 (48.95)		4779 (30.68)	874 (48.95)	
**Current drinking**			<0.001			<0.001
No	144735 (87.91)	6565 (44.60)		13748 (87.45)	752 (42.39)	
Yes	19116 (12.09)	8028 (55.40)		1887 (12.55)	1016 (57.61)	
**Current smoking**			<0.001			<0.001
No	160090 (97.66)	6877 (46.68)		15197 (97.20)	779 (43.81)	
Yes	3761 (2.34)	7716 (53.32)		438 (2.80)	989 (56.19)	
**Subjective healthiness**			<0.001			0.025
Healthy	119873 (72.85)	10683 (73.43)		10012 (63.87)	1135 (64.97)	
Average	34797 (21.43)	2926 (19.80)		4084 (26.32)	431 (23.56)	
Unhealthy	9181 (5.72)	984 (6.78)		1539 (9.81)	202 (11.47)	
**Subjective happiness**			<0.001			<0.001
Happy	110963 (67.23)	8679 (59.25)		10223 (64.67)	977 (55.10)	
Average	41196 (25.52)	4295 (29.31)		3955 (25.87)	528 (29.67)	
Unhappy	11692 (7.25)	1619 (11.44)		1457 (9.46)	263 (15.23)	
**Perceived stress**			<0.001			<0.001
Often	58532 (35.80)	5907 (40.42)		6305 (40.36)	819 (45.80)	
Sometimes	71097 (43.68)	5908 (40.52)		6504 (41.78)	632 (35.55)	
Never	34222 (20.53)	2778 (19.07)		2826 (17.86)	317 (18.65)	
**Sleep satisfaction**			<0.001			<0.001
Plenty	45563 (27.00)	3006 (20.27)		4261 (26.50)	338 (19.13)	
A little	53628 (32.78)	4499 (30.52)		4883 (31.38)	538 (30.76)	
Not enough	64660 (40.22)	7088 (49.22)		6491 (42.12)	892 (50.12)	
**Depression**			<0.001			<0.001
No	125910 (76.76)	9559 (65.26)		11302 (72.09)	1035 (58.21)	
Yes	37941 (23.24)	5034 (34.74)		4333 (27.91)	733 (41.79)	
**Suicidal ideation**			<0.001			<0.001
No	145700 (88.87)	12116 (83.16)		13375 (85.48)	1365 (77.65)	
Yes	18151 (11.13)	2477 (16.84)		2260 (14.52)	403 (22.35)	
**Suicidal attempt**			<0.001			<0.001
No	160463 (97.96)	13810 (94.60)		15167 (96.96)	1581 (89.34)	
Yes	3388 (2.04)	783 (5.40)		468 (3.04)	187 (10.66)	

Numbers represent frequency and column percentages for each characteristic. Current smokers were defined as those who responded ‘more than one day’ to the following question: ‘How many days did you smoke even one cigarette during past 30 days before this survey?’ by the standpoint of the Korea Youth Risk Behavior Web-based Surveys. To measure EC smoking participants were asked a binary (yes or no) question regardless of smoking status or experience: ‘Have you ever used ECs in your lifetime?’. For those who answered yes, a follow-up question was asked: ‘In the last 30 days have you used ECs?’. Other definition or concept of variables are mentioned in the Methods section of the manuscript. Survey data were weighted for statistical representation of the general population based on the sample design. Survey data were weighed for statistical representation of the general population based on the sample design. Chi-squared test was applied to determine statistical differences between categorical data and independent t-test was used for continuous variables.

### Associated factors for depression, suicidal ideation and suicide attempt in asthmatic adolescents with experience of electronic cigarette use

Following adjustment, being female was significantly associated with depression (AOR=1.60; 95% CI: 1.47–1.73), suicidal ideation (AOR=1.34; 95% CI: 1.20–1.49), and suicide attempt (AOR=1.82; 95% CI: 1.50–2.21) (Table 4). Enrollment in high school was inversely associated with depression, suicidal ideation, and suicide attempt. Alcohol consumption and smoking were also associated with depression and suicidality in asthmatic adolescents with experience of EC use. Among several mental health variables, perception of stress was most strongly associated with depression (AOR=4.79; 95% CI: 4.12–5.58). For suicidal ideation and suicide attempt, perception of unhappiness was the strongest association (AOR=5.24; 95% CI: 4.51– 6.09 and AOR=4.37; 95% CI: 3.36–5.69; respectively). EC use itself was significantly associated with asthma (AOR=1.07; 95% CI: 1.01–1.15) (Supplementary file Table S1). Also, EC use was associated with depression (AOR=1.36; 95% CI: 1.15–1.61) and suicidality among asthmatic adolescents with experience of EC use (AOR=1.28; 95% CI: 1.18–1.43 and AOR=2.11; 95% CI: 1.51–2.95).

**Table 4:** Associated factors for depression, suicidal ideation, and suicide attempts in Korean asthmatic adolescents with experience of electronic cigarette use, 2015–2017 (N=17403)

	*Depression*				*Suicidal ideation*				*Suicide attempt*			
	*OR (95% CI)*	*p*	*AOR (95% CI)*	*p*	*OR (95% CI)*	*p*	*AOR (95% CI)*	*p*	*OR (95% CI)*	*p*	*AOR (95% CI)*	*p*
**Sex**												
Female	1.79 (1.67–1.93)	<0.001	1.60 (1.47–1.73)	<0.001	1.56 (1.42–1.71)	<0.001	1.34 (1.20–1.49)	<0.001	1.72 (1.45–2.04)	<0.001	1.82 (1.50–2.21)	<0.001
Male	Ref.				Ref.		Ref.		Ref.		Ref.	
**Grade**												
Middle school	Ref.		Ref.		Ref.		Ref.		Ref.		Ref.	
High school	1.25 (1.16–1.35)	<0.001	0.89 (0.81–0.97)	0.007	1.00 (0.91–1.09)	0.915	0.64 (0.61–0.66)	<0.001	0.80 (0.67–0.96)	0.015	0.45 (0.37–0.55)	<0.001
**SES**												
High	Ref.		Ref.		Ref.		Ref.		Ref.		Ref.	
Middle	0.94 (0.87–1.02)	0.167	0.80 (0.73–0.87)	<0.001	0.87 (0.79–0.96)	0.006	0.76 (0.68–0.85)	<0.001	0.59 (0.48–0.73)	<0.001	0.58 (0.46–0.72)	<0.001
Low	1.61 (1.45–1.78)	<0.001	0.92 (0.82–1.04)	0.170	1.76 (1.57–1.99)	<0.001	0.96 (0.84–1.11)	0.620	1.77 (1.44–2.19)	<0.001	1.01 (0.80–1.28)	0.908
**Academic achievement**												
High	Ref.		Ref.		Ref.		Ref.		Ref.		Ref.	
Middle	1.22 (1.11–1.33)	<0.001	1.21 (1.09–1.33)	<0.001	1.01 (0.90–1.14)	0.851	0.96 (0.84–1.09)	0.523	0.84 (0.66–1.07)	0.162	0.89 (0.70–1.14)	0.370
Low	1.70 (1.57–1.85)	<0.001	1.33 (1.21–1.46)	<0.001	1.48 (1.34–1.64)	<0.001	1.02 (0.91–1.15)	0.721	1.62 (1.33–1.98)	<0.001	1.09 (0.88–1.35)	0.435
**Current drinking**												
No	Ref.		Ref.		Ref.		Ref.		Ref.		Ref.	
Yes	2.02 (1.85–2.21)	<0.001	1.51 (1.34–1.70)	<0.001	1.90 (1.71–2.12)	<0.001	1.34 (1.17–1.54)	<0.001	3.15 (2.63–3.77)	<0.001	1.84 (1.46–2.32)	<0.001
**Current smoking**												
No	Ref.		Ref.		Ref.		Ref.		Ref.		Ref.	
Yes	2.18 (1.93–2.46)	<0.001	1.40 (1.16–1.69)	<0.001	2.17 (1.88–2.50)	<0.001	1.52 (1.22–1.89)	<0.001	4.37 (3.57–5.34)	<0.001	1.95 (1.41–2.71)	<0.001
**Subjective healthiness**												
Healthy	Ref.		Ref.		Ref.		Ref.		Ref.		Ref.	
Average	1.74 (1.60–1.89)	<0.001	1.05 (0.96–1.16)	0.296	1.90 (1.71–2.11)	<0.001	1.09 (0.97–1.23)	0.141	1.80 (1.47–2.20)	<0.001	1.15 (0.92–1.44)	0.205
Unhealthy	2.98 (2.67–3.33)	<0.001	1.30 (1.15–1.48)	<0.001	4.08 (3.59–4.63)	<0.001	1.54 (1.32–1.80)	<0.001	4.21 (3.39–5.24)	<0.001	1.76 (1.38–2.24)	<0.001
**Subjective happiness**												
Happy	Ref.		Ref.		Ref.		Ref.		Ref.		Ref.	
Average	2.41 (2.22–2.62)	<0.001	1.52 (1.38–1.67)	<0.001	2.77 (2.48–3.09)	<0.001	1.79 (1.59–2.02)	<0.001	2.37 (1.90–2.95)	<0.001	1.76 (1.37–2.26)	<0.001
Unhappy	7.35 (6.51–8.29)	<0.001	3.14 (2.73–3.62)	<0.001	12.22 (10.76–13.88)	<0.001	5.24 (4.51–6.09)	<0.001	9.21 (7.46–11.37)	<0.001	4.37 (3.36–5.69)	<0.001
**Perceived stress**												
Often	8.49 (7.35–9.79)	<0.001	4.79 (4.12–5.58)	<0.001	8.67 (7.16–10.50)	<0.001	4.11 (3.35–5.03)	<0.001	3.28 (2.49–4.32)	<0.001	1.44 (1.05–1.99)	0.025
Sometimes	2.18 (1.88–2.52)	<0.001	1.85 (1.60–2.13)	<0.001	1.63 (1.32–2.00)	<0.001	1.37 (1.12–1.68)	0.002	0.68 (0.48–0.95)	0.025	0.64 (0.46–0.90)	0.010
Never	Ref.		Ref.		Ref.		Ref.		Ref.		Ref.	
**Sleep satisfaction**												
Plenty	Ref.		Ref.		Ref.		Ref.		Ref.		Ref.	
A little	1.73 (1.56–1.92)	<0.001	1.32 (0.71–1.29)	<0.001	1.42 (1.24–1.64)	<0.001	1.03 (0.88–1.19)	0.734	1.19 (0.90–1.57)	0.225	0.95 (0.71–1.29)	0.763
Not enough	2.92 (2.65–3.23)	<0.001	1.49 (1.33–1.66)	<0.001	2.84 (2.50–3.22)	<0.001	1.23 (1.06–1.43)	0.006	2.59 (2.04–3.28)	<0.001	1.27 (0.97–1.67)	0.088
**EC use**												
No	Ref.		Ref.		Ref.		Ref.		Ref.		Ref.	
Yes	1.85 (1.66–2.07)	<0.001	1.36 (1.15–1.61)	<0.001	1.69 (1.48–1.94)	<0.001	1.28 (1.18–1.43)	<0.001	3.81 (3.13–4.64)	<0.001	2.11 (1.51–2.95)	<0.001

Following the selection of significant covariates, univariate and multivariate logistic regression analysis were performed to identify the associated factors for depressive mood, suicidal ideation and attempt in asthmatic adolescents with experience of using electronic cigarettes. Odds ratios (OR), adjusted ORs (AORs), and confidence intervals at the 95% level (95% CIs) were obtained.

## DISCUSSION

The main purpose of this study was to identify the EC-experience rate and associated factors for depression and suicidality among Korean asthmatic adolescents who had experience of EC use. We found that, consistent with our hypothesis, rates of current smokers’ experience of EC use and current EC use were more common among asthmatic than non-asthmatic respondents. Our findings add to the available evidence that shows that asthmatic adolescents with experience of EC use have significantly higher risk of psychological distress, including suicidality, compared with adolescents without EC experience. Another noteworthy result of our study is that, among several associated factors, perception of stress was most strongly associated with depression, and perception of unhappiness was most strongly associated with suicidal ideation and suicide attempt. To our knowledge, this is the first study to assess the association of depression and suicidality with experience of EC use, based on a nationally representative sample of Korean asthmatic adolescents.

Although the health effects of ECs are not fully understood and there is no strong evidence that ECs are effective smoking cessation aids^6,14-16^, there has been a rapid increase in EC use, especially among asthmatic adolescents^1,3,6,11-13,30^. There are no data on the potential long-term effects of EC use such as incidence or exacerbation of asthma^6^, however, several studies suggest that adolescent EC use is related to a higher likelihood of having been diagnosed with asthma^1,3,6,12^, and adolescents with asthma have higher odds of smoking ECs than those without asthma^30^. Similarly, the rates of experience of EC use and current EC use were greater in asthmatic than nonasthmatic respondents in our study.

EC use is associated with a less favorable perception of physical health and is associated with increased risk of depressive symptoms and suicidality compared to non-use^20,22,27^. A previous study of US adults showed that current EC users had 2.10 (95% CI: 1.98–2.23) higher odds of reporting a clinical diagnosis of depression than never users^28^. Regarding adolescents, current EC users had 1.58 and 2.44 higher odds, respectively, of suicidal ideation and suicidal attempt compared with non-EC users^20^. Our study revealed a similar basic pattern found in previous studies in that asthmatic respondents with experience of EC use were more likely to have mental health problems, including suicidality, than those without EC experience. Considering that adolescents are more vulnerable to nicotine sensitivity and experience strong emotions and impulsivity because of cognitive immaturity, nicotine exposure through EC use can be especially likely to produce negative consequences of addiction, lower cognitive function, worsen emotion regulation, and maladaptive decisionmaking, and also be associated with depression^20,22^. Internalizing and externalizing disorders were found to be predictors of susceptibility to EC use among US youth^31^. Psychological problems, rebelliousness, ever use of alcohol, marijuana and other substances, and household secondhand smoke exposure were found to be risk factors for increased susceptibility^31^. However, because there might be a bidirectional relationship between ECs and depression, suicidal ideation and suicide attempt, like conventional cigarettes^25^, it is clear that future studies are warranted to investigate the reason for association between mental health problems, including suicidality, and EC use in asthmatic adolescents.

Although not all negative mental health states are connected to suicide attempts, identification of associated factors for suicidality is essential to reduce or prevent suicide. Our study found several associated factors for depression and suicidality among asthmatic respondents with experience of EC use.

First, although males had much higher rates of experience of EC use in our study, females were significantly more likely to report depression and suicidality. This result is similar to findings from studies that suggested the association between EC use and psychological distress may be the same as for conventional cigarettes and psychological distress^22,32^. It is already widely known that female smokers are more likely to suffer from depression and suicidal behavior than male smokers^22^, and females are significantly more likely to attempt suicide due to a sense of helplessness, loneliness, rejection, and conflicts with their parents and peers, than males^26^. However, future studies with greater statistical power should explore the association between suicidality and EC experience among asthmatic adolescents in greater detail, including gender stratification. However, our findings indicate that current suicide prevention interventions should focus on female asthmatic adolescents with experience of EC use.

Second, high school enrollment was inversely associated with depression, suicidal ideation and suicide attempt. These findings may result from the fact that middle school students are more vulnerable than high school students to psychosocial burdens, perhaps because they lack the age-based ability to regulate emotional instability^33^. Because high school students typically have an established sense of selfesteem as well as agency in their own major life decisions (such as choosing a career path or entering college), their risk for depression and suicidal behavior may be lower than for middle school students.

Third, several mental health variables (e.g. stress, feelings of unhappiness) were shown to be significantly associated. Previous studies show differences in mental health conditions between those who use ECs and those who do not; mental health problems are associated with increased risk for EC use^28,34,35^. According to the literature, individuals with mental health problems were more likely to have tried ECs (14.8% vs 6.6%), to be current EC users (3.1% vs 1.1%), and to be susceptible to future EC use (60.5% vs 45.3%) than those without such conditions^35^. EC use is associated with a poor perception of overall health or physical health, poor emotional support, dissatisfaction, feeling of stress, and stigmatization by family or friends^36^. Alternatively, perceived poor physical health or lack of emotional support or feeling stressed may lead some individuals to use ECs^36^.

### Strengths and limitations

This study had several strengths and clinical implications that improve on findings from previous reports. To the best of our knowledge, factors associated with depression and suicidality in asthmatic adolescents with experience of EC use have not been explored with a large population dataset, as we did with this study. We found that gender, school grade, and several mental health measures were strongly associated with depression and suicidality among asthmatic respondents with experience of EC use, and many of these variables had not been investigated in prior research. Furthermore, our study comprised the largest number of participants among similar studies, and was based on a nationwide, government-directed survey with a high response rate (96.3%). Additionally, a socioeconomically diverse sample with an equal proportion of middle school and high school students (400 schools each annually) were represented, and all analyses in this study were based on sample weights and adjusted for the complex sample design of the survey, which allows for generalizability of our findings. The public health implications of our findings can be applied at practical levels. The beginning time for first EC among adolescents with asthma was very early, before middle school (14.5%) and most started in middle or high school in this study. Considering the fact of rapid penetration of ECs in the youth market (the average age of first using ECs decreased from 14.6 years old in 2015 to 13.8 years old in 2017 in Korea)^37^, it is a worrying fact and such information should go to the school and health authorities of the country. Also, in the point of view that the reason of EC use is a strategy used by the tobacco industry in attracting future smokers, respondents cited the availability of appealing flavors, safety, curiosity, and as a smoking cessation aid, as the main reasons for use in this study. Compared to the results in other countries, the reasons for EC use differed according to the study: curiosity, appealing flavors, peer influence, and being able to conceal their use^4^; the buzz (52%), appealing flavors (43%), and peer influence (36%)^38^; flavor availability, social media promotion, discreet flash drive design, and allowing use to go undetected by parents^39^. Public health campaigns and education in schools should target adolescents, especially those with asthma, to raise awareness of the risks of all types of smoking, including ECs. Physicians should be mindful of associated factors for suicide among asthmatic adolescents with experience of EC use. Associated factors, such as being female, enrollment in middle school, alcohol consumption and smoking, and negative mental health, should also be considered. Clinicians, especially pediatric allergists, should actively inquire about current or previous EC use through direct questions or a questionnaire survey when meeting with asthmatic patients, starting with the initial evaluation and throughout follow-up, and they should closely observe any changes in mood.

This study has several limitations. First, this study was cross-sectional in design, so we could not establish causal relationships (in either direction) between influential factors and depression and suicidality in asthmatic adolescents with EC experience. Second, we evaluated only the lifelong diagnosis of asthma in this study, therefore, we could not separate currently active asthma from previous (but treated or inactive) asthma. We did not analyze current symptoms and severity, asthma management, treatment modality, failure of treatment due to potential inadequate medical managements in some cases, and lack of adherence to the proposed treatment because these data were not available from the survey. The proportion of asthma among the overall respondents was 8.9%, which was higher than those of other studies for Korean adolescents^3,11^, probably because of the difference in the definition of asthma (lifelong asthma vs current asthma). This limitation could have led to misclassification of some asthma cases and if these topics were available, some conclusions probably would be changed. Third, the quantity and duration of conventional cigarette or EC consumption were not assessed. Moreover, biochemical analysis of blood or urine cotinine levels was not performed because of the study design. Future prospective follow-up research utilizing a more detailed questionnaire and laboratory testing is necessary^40^. Fourth, because of the self-reported nature of the survey, even though anonymity was guaranteed, some sensitive questions, including those about mental health states and suicidality, may have been influenced by respondent fears that their answers could be linked to private and confidential information, which could have resulted in erroneous reporting, substantially decreased credibility, and potential recall bias. Additionally, because depressive mood was self-reported using a dichotomized response format (‘yes’ or ‘no’), and not depression as diagnosed by a physician, the prevalence of depression may be overestimated or underestimated because of this type of response. Furthermore, we could not analyze data from 3.7% of the targeted subjects who did not participate. If people who did not participate were more likely to have used ECs, our estimates will be biased to some degree. Moreover, because the study population was so enormous, many of the differences emerged as statistically significant, therefore, it should be considered as a limitation. We think a stratified analysis for middle- and high-school students will be meaningful regarding their very different developmental stages. Therefore, another investigation for the comparison of mental health status including depression and suicidality between middle- and high-school students among asthmatic adolescents with experience of EC use is warranted.

## CONCLUSIONS

Although the findings of this study should be considered cautiously, we suggest that many asthmatic adolescents with experience of EC use in Korea have experienced depression and suicidal behaviors. Asthmatic adolescents with depression or suicidality should be carefully evaluated for experience of EC use that may have been previously neglected. Allergists including doctors of adolescents, pulmonologists, family physicians, and other medical specialists, should collaborate with mental health professionals to actively inquire about depression and suicidality in asthmatic adolescents with experience of EC use , especially those with associated factors. Such efforts could potentially reduce suicidality in asthmatic adolescents using ECs.
