# Supplementary material for: Associated factors for depression, suicidal ideation and suicide attempt among asthmatic adolescents with experience of electronic cigarette use
Source: Tob Induc Dis. 2020 Oct 16;18:85. doi: 10.18332/tid/127524 (PMC7586912; doi:10.18332/tid/127524)
Supplement: Supplementary file 1 [file TID-18-85-s1.pdf]

Supplementary Table S1. Associated factors for asthma among 195,847 Korean adolescents from 2015 to 2017

	Crude		Adjusted	
	**OR (95% CI)**	**p-value**	**OR (95% CI)**	**p-value**
**Sex**				
Female	Reference		Reference	
Male	1.35 (1.30-1.40)	<0.001	1.42 (1.67-1.48)	<0.001
**Grade**				
Middle school	1.09 (1.04-1.13)	<0.001	1.17 (1.12-1.22)	<0.001
High school	Reference		Reference	
**Subjective economic state**				
High	Reference		Reference	
Middle	0.90 (0.86-0.93)	<0.001	0.92 (0.88-0.96)	<0.001
Low	1.08 (1.02-1.14)	0.004	1.03 (0.97-1.09)	0.352
**Academic achievement**				
High	Reference		Reference	
Middle	0.91 (0.87-0.95)	<0.001	0.93 (0.89-0.97)	0.001
Low	0.93 (0.89-0.97)	<0.001	0.88 (0.85-0.92)	<0.001
**Current drinking**				
No	Reference		Reference	
Yes	1.11 (1.06-1.16)	<0.001	1.04 (0.99-1.10)	0.129
**Current smoking**				
No	Reference		Reference	
Yes	1.26 (1.18-1.35)	<0.001	1.10 (1.02-1.19)	0.019
**Subjective healthiness**				
Healthy	Reference		Reference	
Average	1.39 (1.34-1.45)	<0.001	1.45 (1.39-1.51)	<0.001
Unhealthy	1.96 (1.84-2.08)	<0.001	1.98 (1.85-2.11)	<0.001
**Subjective happiness**				
Happy	Reference		Reference	
Average	1.06 (1.02-1.10)	0.002	0.94 (0.90-0.98)	0.004
Unhappy	1.38 (1.30-1.47)	<0.001	1.00 (0.94-1.07)	0.991
**Perceived stress**				
Often	1.29 (1.23-1.35)	<0.001	1.24 (1.17-1.31)	<0.001
Sometimes	1.08 (1.03-1.13)	0.002	1.10 (1.05-1.15)	<0.001
Never	Reference		Reference	
**Sleep satisfaction**				
Plenty	Reference		Reference	
A little	0.99 (0.94-1.03)	0.537	0.98 (0.94-1.03)	0.481
Not enough	1.08 (1.03-1.12)	0.001	1.02 (0.98-1.07)	0.322
**EC use**				
No	Reference		Reference	
Yes	1.23 (1.16-1.30)	<0.001	1.07 (1.01-1.15)	0.002

Following the selection of significant covariates, univariate and multivariate logistic regression analysis were performed to identify the associated factors for asthma. Odds ratios (OR), adjusted ORs (aORs), and confidence intervals at the 95% level (95% CIs) were obtained.EC: electronic cigarette.

© 2020 Kim C.W. et al.
